# Incorporation of **Plasmid** DNA **In**to Bacterial **Membrane** Vesicles by **Peptidoglycan Defects** in *Escherichia coli*

**DOI:** 10.3389/fmicb.2021.747606

**Published:** 2021-11-29

**Authors:** Sharmin Aktar, Yuhi Okamoto, So Ueno, Yuhei O. Tahara, Masayoshi Imaizumi, Masaki Shintani, Makoto Miyata, Hiroyuki Futamata, Hideaki Nojiri, Yosuke Tashiro

**Affiliations:** ^1^Department of Engineering, Graduate School of Integrated Science and Technology, Shizuoka University, Hamamatsu, Japan; ^2^Faculty of Engineering, Shizuoka University, Hamamatsu, Japan; ^3^Graduate School of Science, Osaka City University, Osaka, Japan; ^4^The OCU Advanced Research Institute for Natural Science and Technology (OCARINA), Osaka City University, Osaka, Japan; ^5^Graduate School of Science and Technology, Shizuoka University, Hamamatsu, Japan; ^6^Research Institute of Green Science and Technology, Shizuoka University, Shizuoka, Japan; ^7^Agro-Biotechnology Research Center, Graduate School of Agricultural and Life Sciences, The University of Tokyo, Tokyo, Japan; ^8^JST PRESTO, Kawaguchi, Japan

**Keywords:** membrane vesicles, plasmid, peptidoglycan, glycine, quick-freeze deep-etch and replica electron microscopy, outer membrane vesicles

## Abstract

Membrane vesicles (MVs) are released by various prokaryotes and play a role in the delivery of various cell-cell interaction factors. Recent studies have determined that these vesicles are capable of functioning as mediators of horizontal gene transfer. Outer membrane vesicles (OMVs) are a type of MV that is released by Gram-negative bacteria and primarily composed of outer membrane and periplasm components; however, it remains largely unknown why DNA is contained within OMVs. Our study aimed to understand the mechanism by which DNA that is localized in the cytoplasm is incorporated into OMVs in Gram-negative bacteria. We compared DNA associated with OMVs using *Escherichia coli* BW25113 cells harboring the non-conjugative, non-mobilized, and high-copy plasmid pUC19 and its hypervesiculating mutants that included Δ*nlpI*, Δ*rseA*, and Δ*tolA*. Plasmid copy per vesicle was increased in OMVs derived from Δ*nlpI*, in which peptidoglycan (PG) breakdown and synthesis are altered. When supplemented with 1% glycine to inhibit PG synthesis, both OMV formation and plasmid copy per vesicle were increased in the wild type. The bacterial membrane condition test indicated that membrane permeability was increased in the presence of glycine at the late exponential phase, in which cell lysis did not occur. Additionally, quick-freeze deep-etch and replica electron microscopy observations revealed that outer-inner membrane vesicles (O-IMVs) are formed in the presence of glycine. Thus, two proposed routes for DNA incorporation into OMVs under PG-damaged conditions are suggested. These routes include DNA leakage due to increased membrane permeation and O-IMV formation. Additionally, our findings contribute to a greater understanding of the vesicle-mediated horizontal gene transfer that occurs in nature and the utilization of MVs for DNA cargo.

## Introduction

The release of membrane vesicles (MVs) is a widespread phenomenon in prokaryotic cells. These spherical particles are 20–400 nm in a diameter and have been reported to facilitate a number of biological functions, including the delivery of proteins, signals, and toxins to target cells and the release unnecessary substances in the context of the envelope stress response (Tashiro et al., 2012). In particular, MVs derived from Gram-negative bacteria that are also known as outer membrane vesicles (OMVs) are composed of outer membrane (OM) and periplasmic components (Toyofuku et al., 2015).

In bacteria and archaea, genome revolution via recombination with foreign DNA requires horizontal gene transfer (HGT). Vesicle-mediated gene transfer has been identified as a novel mechanism of HGT that functions in addition to the three traditional mechanisms, including conjugation, transformation and transduction (Hasegawa et al., 2015; Tashiro et al., 2019; Soler and Forterre, 2020). Gene exchange via MVs has been observed across a broad range of bacterial species that include Gram-negative bacteria (Dorward et al., 1989; Kolling and Matthews, 1999; Yaron et al., 2000; Rumbo et al., 2011; Fulsundar et al., 2014), Gram-positive bacteria (Klieve et al., 2005) and archaea (Gaudin et al., 2013) that are derived from various environments such as seawater, rumen, gut, and periodontitis (Domingues and Nielsen, 2017). Additionally, bacterial MVs can transfer DNA into the nucleus of eukaryotic cells (Bitto et al., 2017). MV-mediated gene transfer possesses several advantages over other HGT mechanisms. This process protects packaged DNA from degradative enzymes and permits DNA transfer over long distances and time frames. In contrast to transduction with bacteriophages, recipients are not strictly limited in MV-mediated HGT (Tran and Boedicker, 2017). Although plasmid characteristics such as plasmid copy number and origin of replication can affect the efficiency of MV-mediated HGT, this process does not require a donor to possess a specialized set of gene products like conjugation (Tran and Boedicker, 2019).

A variety of Gram-negative bacteria release OMVs, but it remains unclear how DNA localized in the cytoplasm is packaged into OMVs. Proteomic analyses have indicated that cytoplasmic and inner membrane proteins and also periplasmic and outer membrane proteins (OMPs) are all present in OMVs derived from various Gram-negative bacteria (Lee et al., 2007; Choi et al., 2011; Aguilera et al., 2014). A proposed mechanism for the incorporation of DNA into MVs in Gram-negative bacteria is the formation of outer-inner membrane vesicles (O-IMVs) that exist as double membrane structures of OM and inner membrane (IM). These unique MVs were first identified in the ocean bacterium *Shewanella vesiculosa* M7^T^ (Pérez-Cruz et al., 2013). It was subsequently confirmed that O-IMVs are naturally secreted by several Gram-negative bacteria such as *Neisseria gonorrhoeae*, *Pseudomonas aeruginosa*, and *Acinetobacter baumannii*, and DNA and ATP were both detected in the O-IMVs (Pérez-Cruz et al., 2015). Another possible route for the formation of MVs containing cytoplasmic components in Gram-negative bacteria is explosive cell lysis that is triggered by phage-derived endolysin (Turnbull et al., 2016; Toyofuku et al., 2018). Endolysin degrades the peptidoglycan (PG) cell wall to facilitate the formation of MVs that are likely composed of OM or IM. Cell lysis is considered as an underlying reason for the existence of cytoplasmic components such as DNA in MVs. However, it remains unknown how DNA is sorted into OMVs through processes other than the formation of O-IMVs and explosive cell lysis. Additionally, little is known regarding the timing and contributing factors of O-IMV formation.

Currently, several mechanisms have been suggested to be responsible for the blebbing observed in OMV biogenesis based on studies examining mutants that are lacking genes related to bacterial surfaces in Gram-negative bacteria. One of the best-characterized factors is encoded by a gene cluster known as *tol-pal* that is conserved across most Gram-negative bacteria. The Tol-Pal system is comprised of five proteins (TolA, TolB, TolQ, TolR, and Pal), and these components are linked to the inner membrane, PG, and OM (Sturgis, 2001). The deletion of one of these components results in increased vesicle formation due to dissociation of the OM from the underlying PG in various Gram-negative bacteria (Sonntag et al., 1978; Bouveret et al., 1995; Bernadac et al., 1998; Turner et al., 2015; Takaki et al., 2020). The deletion of *nlpI* also induces OMV formation in *E. coli* (McBroom et al., 2006). It has been postulated that the balance between PG breakdown and synthesis is altered in the *nlpI* mutant, and covalent crosslinks are not properly formed between PG and Lpp, which is a major lipoprotein in *E. coli* (Schwechheimer et al., 2015; Schwechheimer and Kuehn, 2015). Additionally, the deletion of σ^E^-related genes, including *rseA*, *degP*, and *degS*, is also known to enhance OMV formation (McBroom et al., 2006). These mutations cause accumulation of misfolded proteins within the periplasm and increase OMV formation in Gram-negative bacteria (McBroom and Kuehn, 2007; Tashiro et al., 2009; Schwechheimer et al., 2014).

In this study, we explored which blebbing mechanism affects the incorporation of DNA into OMVs. We analyzed the DNA content of OMVs derived from an *E. coli* wild type (WT) strain and from several hypervesiculating mutants, including Δ*nlpI*, Δ*rseA*, and Δ*tolA*, using plasmid pUC19. We demonstrated that PG defects increase plasmid sorting into OMVs and that glycine-induced vesicles includes higher concentration of plasmid. Our findings contribute to a greater understanding of how plasmids are contained in OMVs.

## Materials and Methods

### Bacterial Strains, Plasmids, and Growth Conditions

All bacterial strains and plasmids used in this study were listed in Supplementary Table 1. *E. coli* K12 BW25113 (used as WT) and its derivatives (KEIO collection) were obtained from National BioResource Project (National Institute of Genetics) (Baba et al., 2006). Plasmid pCP20 was introduced into each mutant harboring FRT-Km-FRT cassette and the kanamycin (Km) cassette was eliminated using flippase/flippase recognition target (Flp/FRT) recombination. Plasmid pUC19 was introduced into non-marker mutants and the transformants were used for further experiments. *E. coli* cells were grown in lysogeny broth (LB) Miller (1% w/v tryptone, 0.5% w/v yeast extract and 1% w/v NaCl) at 37°C with shaking at 200 rpm or on solid LB agar plates. When required, antibiotics were added to the medium at 100 μg/mL ampicillin or 50 μg/mL kanamycin. 10% glycine was added to be a final concentration of 1%, when necessary.

### Extraction and Quantification of Vesicles

Vesicle extraction from *E. coli* was performed as previously described with some modifications (Ojima et al., 2015). Precultures were inoculated into 100 mL of LB broth with or without 1% glycine at an initial OD_600_ of 0.01, and they were grown with shaking for 16 h at 37°C. Bacterial cells were removed by centrifugation, and the supernatants were filtered through 0.45 μm and 0.2 μm-pore-size filters. Ammonium sulfate was added to the filtrates (final concentration of 400 g/L), and the samples were incubated at room temperature for 1 h. Vesicles were recovered from the suspensions by centrifugation at 9,500 × *g* for 30 min at 20°C. Pellets were resuspended in 15% (v/v) glycerol and concentrated by ultracentrifugation at 150,000 × *g* for 1 h at 4°C using an Himac CP80WX and a P50A3 rotor (Eppendorf Himac Technologies, Hitachinaka, Japan), and they were then resuspended in 50 mM HEPES (pH 6.8)-0.85% (w/v) NaCl (HEPES-NaCl buffer). The vesicles were stored at −80°C until further use.

For quantification of vesicles, extracted vesicles were incubated with 5 μg/mL of FM4-64 for 30 min on a 96-well black plate at 37°C in the dark. The fluorescence intensities of the fluorescently labeled samples were measured using a microplate reader (PerkinElmer, Waltham, MA, United States) at 506/750 nm (excitation/emission wavelength). Water-soluble linoleic acid was used as a standard. Vesicle formation is presented as the amount of vesicles normalized to the cell density.

### Quantification of DNA Concentration

Extracellular and vesicle-associated DNA was quantified using a Quant-iT PicoGreen assay (Thermo Fisher Scientific, Waltham, MA, United States). For quantification of extracellular DNA, cell cultures were centrifugated and the supernatants were filtered through 0.45 μm and 0.2 μm-pore-size filters to remove bacterial cells. If necessary, vesicle-free supernatants were prepared by the ultracentrifugation at 150,000 × *g* for 1 h at 4°C. DNA concentration of the supernatant was measured using the PicoGreen. A Tris-EDTA (TE) buffer (pH 8.0) was used to dilute the PicoGreen and lambda-DNA. A standard curve was constructed by serial dilution of DNA based on quantitation by the commercial provider and then incubated for 5 min at room temperature while protected from light. After incubation, the sample fluorescence was measured using a microplate reader (PerkinElmer) at 480 nm for excitation and 520 nm for emission.

For quantification of vesicle-associated DNA, extracted vesicles were treated with GES lysis reagent (5M guanidinum thiocyanate, 100 mM EDTA, 0.5% w/v sodium *N*-lauroylsarcosinate) for prior to labeling. DNA concentrations were measured by PicoGreen, and the values were normalized to the vesicle protein concentration. Vesicles were lysed with 1% (w/v) SDS and protein concentration of vesicles were determined using a Micro BCA Protein Assay Reagent Kit (Thermo Fisher Scientific).

Internal vesicle-associated DNA was obtained by the treatment of vesicles (20 μg protein) with 2 U DNase I (Fujifilm Wako Pure Chemical Co., Osaka, Japan) at 37°C for 30 min according to the manufacturer’s directions, followed by release of the internal DNA by lysis of vesicles with GES reagent. External and internal DNA associated with vesicles derived from *E. coli* grown with and without 1% glycine was quantified using PicoGreen.

### Particle Analyses

Hydrodynamic diameters of vesicles were measured by a Zetasizer Nano ZS particle analyzer (Malvern Panalytical Ltd., Malvern, United Kingdom) in HEPES-NaCl buffer at 30°C. The hydrodynamic zeta average diameter was calculated using the dynamic light scattering (DLS) method. The concentration of vesicles was measured by nanoparticle tracking analysis (NTA) using a NanoSight LM10 instrument (Malvern) equipped with an sCMOS camera (Andor, Belfast, United Kingdom) as described previously (Tashiro et al., 2017). Briefly, the samples were diluted in HEPES-NaCl buffer, five replicate videos were collected from each sample, and particle movement was analyzed using NTA software (Version 3.1, Malvern). The velocity of particle movement was used to calculate the particle size according to the two-dimensional Stokes-Einstein equation.

### Quantitative PCR Analyses

The plasmid copy number was determined by real-time PCR analysis. For quantification of internal vesicle-associated plasmid, extracted vesicles were treated with DNase I at 37°C for 30 min to remove external DNA, DNase I was inactivated at 75°C for 15 min, and internal DNA was released from vesicles at 100°C for 10 min. DNA fragments were amplified and quantified using LightCycler FastStart DNA Master SYBR Green I (Roche, Basel, Switzerland) with specific primer pairs (M13-F/M13-R for pUC19 and dxs-F/dxs-R for chromosome listed in Supplementary Table 1) on a LightCycler 2.0 (Roche) according to the manufacturer’s instructions.

### Membrane Permeability Assays

The permeability of bacterial membrane was analyzed by using *Bac*Light Live/Dead bacterial viability staining kit (Thermo Fisher Scientific). Bacterial cells grown at the late exponential phase was centrifuged and washed twice with 0.85% NaCl. Cell suspension was treated with SYTO9 and propidium iodide (PI) following the manufacturer’s instruction. Cells were observed using the Olympus IX73 (Olympus, Tokyo, Japan) microscope, and images were captured with the charge-coupled-device (CCD) camera DP73 and processed by the imaging software cellSens.

The permeability of the outer membrane was analyzed by using fluorescent probe 1-*N*-phenylnaphthylamine (NPN) as previously described (Soh et al., 2020). Briefly, bacterial cells were centrifuged and washed twice with 5 mM HEPES buffer (pH 7.2) containing 1 mM sodium azide. Cell suspension was adjusted to OD_600_ = 0.5 and kept at room temperature for 30 min. 200 μL of samples were applied to black microtiter plate and 4 μL of 500 mM NPN was mixed to achieve a final concentration of 10 μM. The NPN fluorescence was measured using a microplate reader (PerkinElmer) at 350 nm for excitation and 420 nm for emission.

### Transmission Electron Microscopic Observation

For observation of negatively stained vesicles, samples were placed on Cu400 mesh grids (JEOL, Tokyo, Japan) that were pretreated with 0.01% α-poly-L-lysine. Bacterial cells and vesicles were stained with 2% (NH_4_)_6_Mo_7_O_24_ and observed using a JEM-1010 (JEOL) at 80 kV that was equipped with a FastScan-F214 (T) CCD camera (TVIPS, Gauting, Germany).

For quick freeze and replica electron microscopic (QFDE-EM) observations, the protocol was as described previously (Takaki et al., 2020). Briefly, bacterial cells were centrifuged, washed, mixed with a rabbit lung slab and mica flakes, and then placed on a paper disk attached to an aluminum disc. The samples were quickly frozen in liquid helium using a CryoPress (Valiant Instruments, St. Louis, MO, United States). The specimens were placed in a chamber maintained at −180°C using a JFDV freeze-etching device (JEOL). The samples were freeze-fractured with a knife and freeze-etched. Subsequently, the samples were coated with platinum and then coated with carbon. The replicas were floated in full-strength hydrofluoric acid, rinsed in water, cleaned with a commercial bleach containing sodium hypochlorite, and rinsed in water. Replica specimens were placed onto grids and observed using a JEM-1010 (JEOL).

## Results

### Incorporation of Plasmid Into Vesicles Is Increased in *nlpI* Mutant

**FIGURE 1:**
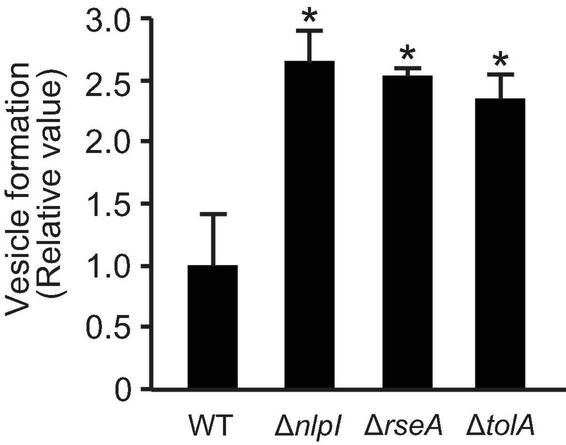
Vesicle formation from *E. coli* BW25113 and mutants. WT, Δ*nlpI*, Δ*rseA*, and Δ*tolA*, all harboring plasmid pUC19, were grown in LB medium containing ampicillin overnight. The amount of vesicles extracted from the supernatants was normalized to cell density, and each value shown is relative to that of WT. The data are presented as the mean ± standard deviation from three replicates. *P < 0.05 compared to WT.

The incorporation of plasmid DNA into OMVs has previously been reported in *E. coli* (Tran and Boedicker, 2017, 2019); however, the mechanism by which plasmid DNA moves from the cytoplasm to OMVs has not been fully elucidated. To gain further insight regarding this process, we focused our studies on examining the relationship between OMV biogenesis and the incorporation of plasmids into MVs. We used *E. coli* BW25113 WT and three high OMV-producing strains (Δ*nlpI*, Δ*rseA*, or Δ*tolA*) that possessed differing hypervesiculating mechanisms. The non-conjugative, non-mobilized and high copy number plasmid pUC19 was used in this experiment, as the copy number per vesicle was the highest among plasmids as previously tested by Tran and Boedicker (2017). These mutants harboring pUC19 exhibited higher OMV formation compared to that of the control strain WT/pUC19 (Figure 1). The average hydrodynamic diameter of OMVs derived from Δ*nlpI* was slightly less than that from WT, and those from Δ*rseA* and Δ*tolA* did not differ from that of WT (Supplementary Figure 1).

**FIGURE 2:**
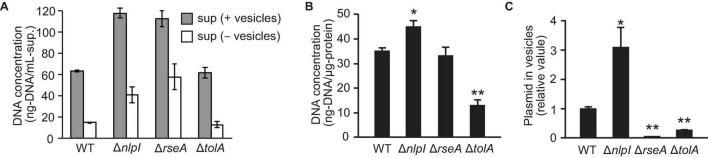
DNA associated with vesicles. *E. coli* BW25113 WT, Δ*nlpI*, Δ*rseA*, and Δ*tolA*, all harboring pUC19, were grown overnight in LB medium containing ampicillin. **(A)** The extracellular DNA (eDNA) concentration of the supernatant (sup.) with and without vesicles was measured by PicoGreen. **(B)** DNA associated with vesicles derived from *E. coli*. Vesicles were lysed with guanidium thiocyanide prior to labeling. DNA concentrations were measured by PicoGreen, and the values were normalized to the vesicle protein concentration. **(C)** The ratio of plasmid to DNase I-treated vesicles. The amount of plasmid and vesicles were measured by real-time PCR and nano-tracking analysis, respectively, and the values (plasmid/vesicle) relative to control (WT/pUC19) are provided. In all figures, the data are presented as the mean ± standard deviation from three replicates. *P < 0.05; **P < 0.005 compared to WT.

To investigate the association of DNA with OMVs, DNA concentration was investigated using the PicoGreen assay that detects double-stranded DNA. First, we measured the DNA concentration of the supernatant before and after ultracentrifugation to examine the extent of DNA associated with OMVs. Concentrations of extracellular DNA (eDNA) in Δ*nlpI* and Δ*rseA* were higher than those in WT, while the eDNA concentration was significantly decreased in the OMV-free supernatant (Figure 2A), suggesting that most of eDNA is associated with OMVs. Next, we investigated the DNA concentration associated with the extracted OMVs. As PicoGreen reagent is not permeable through the cellular membrane in the absence of additional treatments, OMVs were lysed with guanidium thiocyanide prior to labeling with PicoGreen. The DNA associated with OMVs derived from Δ*nlpI* was slightly increased compared to levels derived from WT (Figure 2B), thus indicating that the total of external and internal DNA associated with OMVs was highest in OMVs from Δ*nlpI.*

To analyze the plasmid DNA concentration in OMVs, extracted OMVs were treated with DNase I to remove externally associated DNA from OMVs, and the ratio of plasmid to vesicle was evaluated using real-time PCR and nano-tracking analysis. The amount of plasmid DNA in vesicles was increased threefold in Δ*nlpI*/pUC19 and was significantly decreased in Δ*rseA*/pUC19 and Δ*tolA*/pUC19 compared to that in WT/pUC19. By contrast, the relative plasmid copy number in bacterial cells was not significantly different among the samples (Supplementary Figure 2). These results suggest that defects in peptide crosslinks in PG can increase the incorporation of plasmids into OMVs in Δ*nlpI* even though the average size of these OMVs was smaller than that of the OMVs from the other strains.

### Glycine Enhances the Vesicle Release in *E. coli* BW25113

**FIGURE 3:**
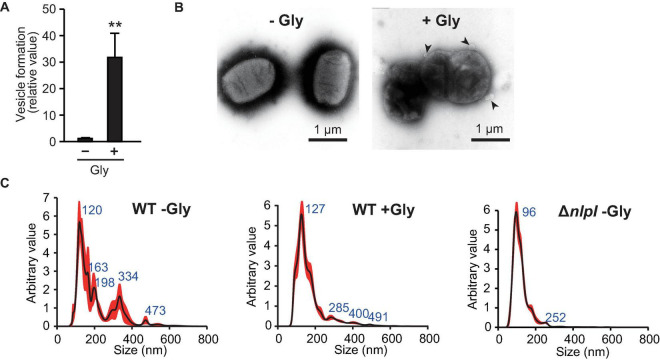
Vesicle formation was increased by glycine. **(A)** Vesicle formation of *E. coli* BW25113/pUC19 in the absence (-Gly) and presence (+ Gly) of 1% glycine. The amount of vesicles extracted from the supernatants was normalized to the cell density, and each value presented is relative to that of control. **P < 0.005 compared to WT. **(B)** *E. coli* BW25113 grown with and without 1% glycine was negative-stained and observed by transmission electron microscopy. Black arrows indicate vesicles. Scale bars are 1 μm. **(C)** Nano-tracking analysis of vesicles from *E. coli* BW25113 WT (with and without glycine) and Δ*nlpI*. Blue numbers indicate the size (nm) of peaks. Each sample was measured three times.

To understand the relationship between defects in peptide crosslinks in PG and the association of DNA with OMVs, we investigated the impact of glycine on OMV formation. Glycine inhibits PG synthesis by substituting glycine for the D- or L-alanine of PG during growth in *E. coli* (Hammes et al., 1973), and the addition of glycine significantly enhances OMV formation in *E. coli* Nissle 1917 (Hirayama and Nakao, 2020). As described in a previous report, the addition of a final concentration of 1% (w/v) glycine enhanced OMV formation in *E. coli* BW25113 harboring pUC19 (Figure 3A). The presence of OMVs surrounding bacterial cells with 1% glycine was confirmed by TEM (Figure 3B), which are thought to be vesicles that did not leave the cell surface due to increased formation. The size distribution of OMVs was not significantly different between OMVs in the presence and absence of 1% glycine, although OMVs derived from Δ*nlpI*/pUC19 did exhibit smaller diameters (Figure 3C).

### Glycine Promotes the Incorporation of DNA Into Vesicles

**FIGURE 4:**
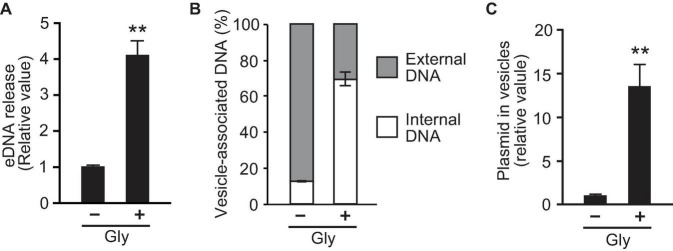
Glycine induces the incorporation of plasmid into vesicles. **(A)** The production of extracellular DNA (eDNA) in *E. coli* BW25113 in the absence (-Gly) and presence (+ Gly) of 1% glycine. DNA concentration in the supernatant was normalized to cell densities, and values relative to that of control are presented. **(B)** Percentage of external and internal DNA of vesicles from *E. coli* BW25113. Vesicles were treated with or without DNase I, and the DNA concentration was measured using PicoGreen. **(C)** The ratio of plasmid to DNase I-treated vesicles. The amount of plasmid and vesicles were measured by real-time PCR and nano-tracking analysis, respectively, and the values (plasmid/vesicle) relative to control (-Gly) are provided. In all figures, the data are presented as the mean ± standard deviation from three replicates. **P < 0.005 compared to the control.

To investigate the association between glycine-induced PG deficiency and DNA secretion, we examined the DNA content within the supernatant and in OMVs. The amount of eDNA released per cell was quantified using the PicoGreen assay and according to cellular density, and the results revealed that eDNA release in the presence of 1% glycine was fourfold higher than that in the control (Figure 4A). To compare DNA content inside and outside of OMVs, DNA concentrations were measured in OMVs treated with or without DNase. The percentage of internal DNA relative to total DNA associated with OMVs was 13% in OMVs derived from *E. coli* in LB medium and 70% in OMVs with glycine (Figure 4B), thus suggesting that the addition of glycine enhances the amount of internal DNA in OMVs. Furthermore, real-time PCR analysis indicated that the ratio of plasmid to OMVs induced by glycine were higher than were those in the control (Figure 4C). These results support the idea that PG defects increase the incorporation of plasmid DNA into OMVs.

### Peptidoglycan Defects Increases Membrane Permeability

**FIGURE 5:**
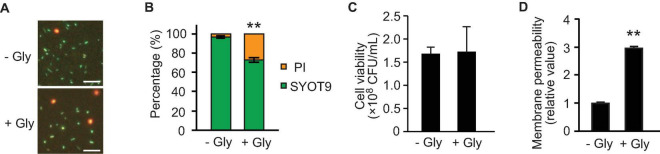
Effect of glycine on membrane permeability. *E. coli* BW25113 was grown to the late exponential phase in the presence and absence of 1% glycine. **(A)** Membrane permeability analyses using SYTO9 and propidium iodide (PI). Bacterial cells exhibiting membrane integrity and deficiency were labeled green and red, respectively. Representative images are presented. Bar = 100 μm. **(B)** The percentages of SYTO9- and PI-labeled cells. The data are presented as the mean ± standard deviation from more than three representative images. **P < 0.005 compared to the control (-Gly). **(C)** Cell viability in the presence and absence of glycine. Bacterial cells were adjusted to equal optical densities (OD_600_ = 1.0), diluted samples were plated onto LB agar, and colony forming units (CFUs) were calculated. The data are presented as the mean ± standard deviation from three replicates. **(D)** The permeability of the outer membrane was assessed by measuring the fluorescence of 1-*N*-phenylnaphthylamine (NPN), and the values relative to that of control are presented. The data are presented as the mean ± standard deviation from three replicates. **P < 0.005 compared to the control (-Gly).

As glycine inhibits the proper alignment of PGs in *E. coli* (Hammes et al., 1973; Jonge et al., 1996), we evaluated the growth and membrane integrity of *E. coli* in the absence and presence of glycine. We analyzed membrane permeability using SYOT9 and PI, both of which are frequently used to test bacterial viability. The growth curve revealed that the presence of 1% glycine slightly repressed *E. coli* growth in LB medium (Supplementary Figure 3). At the late exponential phase (3 h culture), the percentage of PI-labeled cells was increased (Figures 5A,B); however, the colony forming unit (CFU) did not change significantly in the presence or absence of glycine (Figure 5C), suggesting that glycine increases the membrane permeability.

To further analyze the effect of glycine on membrane permeability, we used the fluorescent probe 1-*N*-phenylnaphthylamine (NPN) that is a small hydrophobic molecule (219 Da) and cannot cross the OM. When the membrane is damaged, the hydrophobic molecule NPN can enter into the phospholipid layer, resulting in prominent fluorescence (Muheim et al., 2017). At the late exponential phase, the membrane permeability of OM showed a high level (approximately threefold) in the presence of 1% glycine (Figure 5D). Thus, membrane remodeling already occurred at the late exponential phase, although the bacterial viability was not altered at that point.

### Visualization of Released Vesicles and Cellular Surface Exposure to Glycine

**FIGURE 6:**
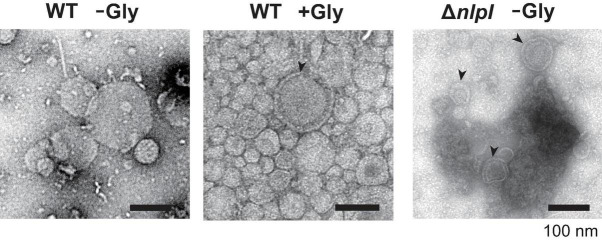
Transmission electron microscopy observation of vesicles. *E. coli* BW25113 WT and Δ*nlpI* harboring pUC19 were grown in LB containing ampicillin with or without 1% glycine, and vesicles extracted from the supernatant were observed after negative staining. Bar = 100 nm.

O-IMVs have been suggested as a route for MV-mediated gene transfer in several bacterial species (Pérez-Cruz et al., 2013, 2015). To evaluate the relationship between DNA incorporation and the appearance of OMVs, OMVs derived from WT (with and without 1% glycine), and Δ*nlpI* were analyzed by negative staining using transmission electron microscopy (TEM). Most OMVs were single spherical vesicles in all samples; however, double lamellar vesicles were observed in OMVs of WT grown with glycine or from Δ*nlpI* (Figure 6 and Supplementary Figure 4). This suggested that these double lamellar vesicles are O-IMVs and may contain cytoplasmic components, including DNA.

**FIGURE 7:**
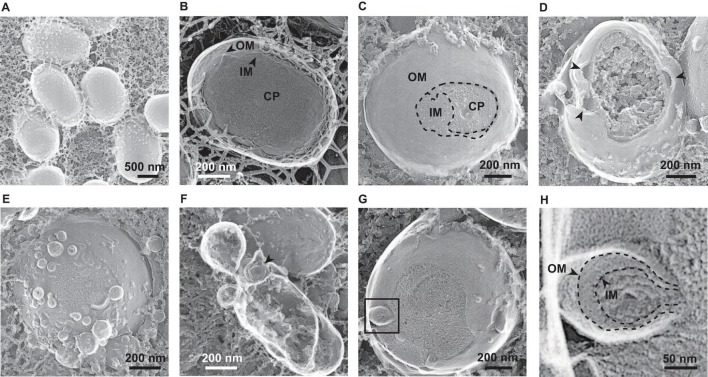
Visualization of bacterial surfaces by quick freeze deep-etch and replica electron micrograph (QFDE-EM) analyses. *E. coli* BW25113 was grown in LB in the absence **(A,B)** and presence **(C–H)** of 1% glycine. The outer membrane (OM), inner membrane (IM), and cytoplasm (CP) are shown **(B,C)**. The black arrows indicate loose and curved OM portions **(D)** and double lamellar vesicles **(F)**, respectively. Enlarged image of the black box in image **(G)** are shown in image **(H)**.

To investigate the spatial structure of the bacterial envelope after exposure to glycine, we used quick-freeze deep-etch and replica electron microscopy (QFDE-EM) to observe bacterial cellular surface with high spatial and sub-millisecond time resolutions (Tulum et al., 2019; Takaki et al., 2020). When *E. coli* cells were grown without glycine, abnormally altered cellular surfaces were not observed in the freeze-fractured sections (Figures 7A,B). The QFDE-EM method enables visualization of the intracellular compartments such as the presumptive OM, IM, and cytoplasm (CP) (Figures 7B,C). The addition of glycine caused the separation of OM and IM and resulted in curvature of the OM (Figure 7D). Furthermore, a large amount of OMVs were observed on the cellular surface in the presence of glycine (Figure 7E). Interestingly, vesicles composed of double membranes were observed in the sample containing glycine (Figure 7F). The freeze-fractured section of the blebbing cells revealed that the multilamellar vesicles were O-IMVs and contained cytoplasmic components (Figures 7G,H), thus suggesting that O-IMVs are a possible route for incorporation of DNA into OMVs in the presence of glycine.

## Discussion

A number of previous studies have reported the presence of DNA in OMVs in several Gram-negative bacteria (Dorward et al., 1989; Dorward and Garon, 1990; Kadurugamuwa and Beveridge, 1995), and OMV-mediated gene exchange has been extensively investigated (Domingues and Nielsen, 2017; Soler and Forterre, 2020). Although vesicle-mediated gene transfer is estimated to occur at a low rate compared to that of the other three HGT mechanisms (transformation, transduction, and conjugation) according to mathematical models (Nazarian et al., 2018), this process possesses unique characteristics compared to those of the others; where specific genes such as *dprA* and *com* operon for natural transformation, phage genes for transduction, and type IV secretion system genes (relaxase, type IV coupling protein, and type IV secretion system) for conjugation are not required. To use vesicles as DNA cargo among bacterial species, the general mechanism of gene exchange has recently been studied using OMVs from *E. coli* (Tran and Boedicker, 2017, 2019). In contrast, much less is known regarding the mechanism by which DNA passes through the inner membrane and is sorted into OMVs in Gram-negative bacteria. In the present study, we explored factors that influence DNA packing into vesicles using OMV-overproducing *E. coli* mutants, and showed that PG defects increase the incorporation of plasmid DNA into OMVs. Furthermore, glycine was identified as a stimulator for OMV production and incorporation of plasmid DNA into OMVs.

In this study, the presence of plasmid DNA in OMVs was examined using hypervesiculating *E. coli* mutants harboring high-copy plasmids. Tran and Boedicker (2017) reported that an average of 3.62 pUC19 plasmids were loaded per *E. coli* vesicle. However, both their and our experiments evaluated the relative ratio of the specific region of the plasmid to that of chromosome, and we cannot debate the absolute copy number of plasmid per vesicle from those results. Further quantitative analyses are required to estimate the copy number present inside the vesicles. As the radius of gyration of pUC19 was estimated to be 65.6 nm (Störkle et al., 2007), several copies at a maximum are presumed to be contained per vesicle. In our results, the loading of pUC19 into vesicles was increased by approximately threefold in the Δ*nlpI* in which peptide crosslinks were defective in PGs (Figure 2C). Interestingly, OMVs from Δ*nlpI* possessed small diameters according to both hydrodynamic (Supplementary Figure 1) and nano-tracking (Figure 3C) analyses despite harboring higher plasmid copies. The PG defects disturb OM-PG links, and OMVs derived from the cellular surface through such mechanisms tend to exhibit smaller diameters compared to those of OMVs derived from cell-binding sites (Deatherage et al., 2009). Thus, we speculated that the incorporation of DNA into OMVs is driven by PG defects.

**FIGURE 8:**
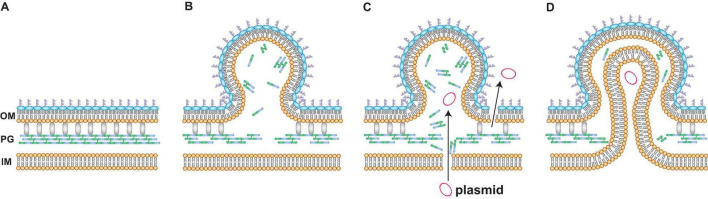
A possible model for DNA incorporation into vesicles. **(A)** The spatial image of the surface of *Escherichia coli*. Peptidoglycan (PG) is localized between the outer membrane (OM) and the inner membrane (IM). Braun’s lipoprotein Lpp (gray) forms crosslinks between OM and PG. **(B)** Defects in PG increase outer membrane vesicle (OMV) formation. The depletion of NlpI or the addition of glycine causes defects in peptide crosslinks in PG. The loss of bridges between OM and PG and the accumulation of PG fragments increase OM curvature, ultimately resulting in OMV formation. **(C,D)** Mechanisms for the incorporation of plasmids into OMVs in *E. coli* cells after exposure to glycine are explained by two proposal models. One is the leakage of plasmids **(C)**. The membrane permeabilities of both OM and IM are increased when PG is not synthesized normally. The other mechanism is the formation of outer-inner membrane vesicles (O-IMVs) **(D)**. IM and cytoplasmic components such as plasmids are included in the vesicle that is leaving the cell. The encapsulation of IM is considered to occur at the site where peptidoglycan is not normally synthesized.

To further corroborate if PG defect-based OMV production enhances the encapsulation of plasmid DNA into OMVs, the influence of 1.0% glycine supplementation on plasmid copy numbers within OMVs was investigated. Glycine substitutes for D- and L-alanine of PGs during growth (Hammes et al., 1973; Jonge et al., 1996), and it also induces OMV production (Hirayama and Nakao, 2020). The mechanism for OMV induction by glycine is considered to be a similar phenomenon to that observed in the depletion of *nlpI*, where the loss of the OM-PG bridge (i.e., Braun’s lipoprotein Lpp) increases OM looseness and the accumulation of PG fragments increases OM protrusion, thus resulting in OMV production (Figures 8A,B). Our data demonstrated that supplementation with 1% glycine enhanced both OMV and eDNA release (Figures 3A, 4A). Additionally, approximately 70% of the DNA associated with OMVs from *E. coli* treated with glycine was the internal DNA in OMVs (Figure 4B). Notably, glycine-induced OMVs contained 13-fold higher pUC19 copies per vesicle compared to those without glycine (Figure 4C). Therefore, glycine can function as an effective additive for producing plasmid-containing OMVs.

Two mechanisms for the incorporation of plasmid DNA into OMVs under PG-defective conditions were proposed in this study. One mechanism is the leakage of DNA from the cytoplasm. Our data revealed that glycine increased the percentage of PI-labeled cells despite no differences in CFUs at the late exponential phase (Figures 4A, 5D), thus indicating that glycine enhances membrane permeability prior to the occurrence of cell lysis. Moreover, eDNA release and OM permeability were increased by glycine (Figures 5A,D). Taken together, our results indicated that cytoplasmic plasmid DNA is leaked into the periplasmic space and the extracellular milieu, ultimately leading to the incorporation of DNA into OMVs and eDNA release (Figure 8C). While PI is permeable to pores that are approximately 1.5 nm in diameter (Nesin et al., 2011), much larger pores could contribute to plasmid DNA penetration. The effect of glycine on PG weakness has been used for effective transformation in several previous studies (Holo and Nes, 1989; Thompson and Collins, 1996; Buckley et al., 1999; Ito and Nagane, 2001), and our results are consistent with those from previous reports indicating that PG defects contribute to DNA permeation through cellular membranes. The internal existence of double-stranded DNA in a single OM bilayer vesicle has also been reported in *Acinetobacter baylyi* (Fulsundar et al., 2014) and *P. aeruginosa* (Bitto et al., 2017), and increased membrane permeability may be one of the proposed mechanisms underlying these observations. It is not yet clear to what extent the existence of vesicles containing interior DNA is due to elevated IM permeability, and quantitative analyses will allow for improved understanding of vesicle-mediated gene transfer in future studies.

The other proposed mechanism for DNA incorporation into OMVs is the production of O-IMVs. The O-IMV production is a natural event for the growth of pathogenic bacteria, including *N. gonorrhoeae*, *P. aeruginosa* and *A. baumannii* (Pérez-Cruz et al., 2015). In our results, O-IMVs were observed in OMVs from Δ*nlpI* and WT supplemented with glycine, but not in those from WT without glycine (Figure 6 and Supplementary Figure 4). When PG is not synthesized accurately, IM can become protruded and encapsulated into OMVs (Figure 8D). The presumption is also supported by a recent report; double lamellar vesicle formation is promoted in a hypervesiculating *E. coli* mutant Δ*mlaE*Δ*nlpI* (Ojima et al., 2021), in which phospholipids are accumulated at the OM in the Δ*nlpI* background (Ojima et al., 2020). Intracellular vesicles, considered to be derived from IM, are accumulated in the periplasmic spaces and finally multilamellar vesicles are formed in the Δ*nlpI* and Δ*mlaE*Δ*nlpI* mutants. We have recently observed excessive intracellular vesicle accumulation in the periplasm and multilamellar/multivesicular outer membrane vesicle (M-OMV) formation in *Buttiauxella agrestis* Δ*tolB* mutant (Takaki et al., 2020), but it is still unclear whether common factors are related to in M-OMV and O-IMV formations. MV formation beyond the PG also occurs in Gram-positive bacteria, where MVs are pinched off from the sites where PG is damaged by the expression of a prophage-encoded endolysin in *Bacillus subtilis* (Toyofuku et al., 2017). As IM exists within the PG in Gram-negative bacteria, an analogous principle may exist in Gram-negative bacterial O-IMV formation and Gram-positive MV formation.

Damage to PG frequently occurs in nature due to incomplete PG synthesis or PG degradation. PG-hydrolyzing enzymes are widespread and classified as endolysins, exolysins, and autolysins based on their origin and role (Vermassen et al., 2019). These PG hydrolyzing enzymes are used for cell division, lytic bacteriophage, and several types (type II, IV, and VI) of secretion systems. Indeed, the existence of autolysins has been confirmed in MVs from *P. aeruginosa*, and endolysin is an endopeptidase that cleaves a critical amide bond between the glycan moiety and the peptide moiety of the PG (Kadurugamuwa and Beveridge, 1995; Li et al., 1996). Therefore, PG damage-based incorporation of plasmid DNA into OMVs has potentially occurred during normal growth.

## Conclusion

We demonstrated that PG defects contribute to the incorporation of DNA into OMVs in addition to OMV formation in *E. coli*. Glycine may provide a useful tool for the development of DNA-containing vesicles for genetic manipulation. Encapsulation of DNA in OMVs under PG defects is considered to occur by two proposed mechanisms that include increased membrane permeability and O-IMV formation. The results of this study are important for providing a better understanding of vesicle-mediated HGT in the nature and the utilization of vesicles for DNA cargo.

## Data Availability Statement

The authors acknowledge that the data presented in this study must be deposited and made publicly available in an acceptable repository, prior to publication. Frontiers cannot accept a manuscript that does not adhere to our open data policies.

## Author Contributions

MS, HN, and YT planned and designed the experiments. SA, YO, and MI extracted vesicles, quantified DNA, and analyzed the vesicles. SU, YOT, and MM contributed to the electron microscopic analyses. HF and YT supervised. SA, YO, and YT wrote the manuscript. All authors critically reviewed the manuscript, analyzed, and discussed the data.

## Conflict of Interest

The authors declare that the research was conducted in the absence of any commercial or financial relationships that could be construed as a potential conflict of interest.

## Publisher’s Note

All claims expressed in this article are solely those of the authors and do not necessarily represent those of their affiliated organizations, or those of the publisher, the editors and the reviewers. Any product that may be evaluated in this article, or claim that may be made by its manufacturer, is not guaranteed or endorsed by the publisher.
