# Supplementary material for: Incorporation of Plasmid DNA Into Bacterial Membrane Vesicles by Peptidoglycan Defects in Escherichia coli
Source: Front Microbiol. 2021 Nov 29;12:747606. doi: 10.3389/fmicb.2021.747606 (PMC8667616; doi:10.3389/fmicb.2021.747606)
Supplement: Supplementary file 1 [file Data_Sheet_1.pdf]

Supplementary Information

Incorporation of plasmid DNA into bacterial membrane vesicles by peptidoglycan defects in *Escherichia coli*.

Sharmin Aktar[1]†, Yuhi Okamoto[2]†, So Ueno[1], Yuhei O Tahara[3,4], Masayoshi Imaizumi[2], Masaki Shintani[1,2,5,6], Makoto Miyata[3,4], Hiroyuki Futamata[1,2,5,6], Hideaki Nojiri[7], Yosuke Tashiro[1,2,5,8]*

[1] Department of Engineering, Graduate School of Integrated Science and Technology, Shizuoka University, Hamamatsu, 432-8561, Japan

[2] Faculty of Engineering, Shizuoka University, Hamamatsu, 432-8561, Japan

[3] Graduate School of Science, Osaka City University, Osaka 558-8585, Japan.

[4] The OCU Advanced Research Institute for Natural Science and Technology (OCARINA), Osaka City University, Osaka 558-8585, Japan

[5] Graduate School of Science and Technology, Shizuoka University, Hamamatsu 432-8561, Japan

[6] Research Institute of Green Science and Technology, Shizuoka University, Suruga-ku, Shizuoka 422-8529, Japan

[7] Agro-Biotechnology Research Center, Graduate School of Agricultural and Life Sciences, The University of Tokyo, Tokyo, Japan

[8] JST PRESTO, Kawaguchi, Saitama, 332-0012, Japan

*** Correspondence:**
Corresponding Author
tashiro.yosuke@shizuoka.ac.jp
† These authors have contributed equally to this work and share first authorship.

Keywords: membrane vesicles, plasmid, peptidoglycan, glycine, quick-freeze deep-etch and replica electron microscopy.

Table S1 Strains, plasmids and primers used in this study.

Strains and plasmids	Genotype	References
Escherichia coli		
DH5α	F^-, Φ80d*lacZ*ΔM15, Δ(*lacZYA-argF*)U169, *deoR*, *recA*1, *endA*1, *hsdR*17(r_K^-, m_K^+), *phoA*, *supE*44, λ^-, *thi*-1, *gyrA*96, *relA*1	Laboratory stock
BW25113	*rrnB, Δ(araD-araB)567,* Δ*lacZ4787(::rrnB-3),* λ^-, *rph-1*, *Δ(rhaD-rhaB)568*, *hsdR514*	(Baba et al., 2006)
JW0729	BW25113 Δ*tolA::FRT-Km-FRT*	(Baba et al., 2006)
JW2556	BW25113 Δ*rseA::FRT-Km-FRT*	(Baba et al., 2006)
JW3132	BW25113 Δ*nlpI::FRT-Km-FRT*	(Baba et al., 2006)
Δ*tolA*	BW25113 Δ*tolA* derived from JW0729	This study
Δ*rseA*	BW25113 Δ*rseA* derived from JW2556	This study
Δ*nlpI*	BW25113 Δ*nlpI* derived from JW3132	This study
Plasmids		
pUC19	High copy cloning vector, pMB1, *bla*	Laboratory stock
pCP20	Flp recombinase, Cm^R	(Datsenko and Wanner, 2000)
Primers		
M13-F	5'-GTAAAACGACGGCCAG-3'	(Ge et al., 2014)
M13-R	5'-CAGGAAACAGCTATGAC-3'	(Ge et al., 2014)
dxs-F	5'-CGAGAAACTGGCGATCCTTA-3'	(Lee et al., 2006)
dxs-R	5'-CTTCATCAAGCGGTTTCACA-3'	(Lee et al., 2006)

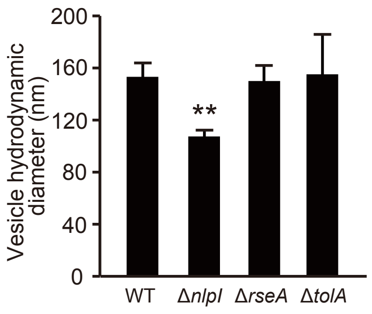

Supplementary Figure 1. Hydrodynamic diameters of vesicles. WT, Δ*nlpI*, Δ*rseA*, and Δ*tolA*, harboring plasmid pUC19 was grown in LB medium containing ampicillin overnight, and vesicles were extracted from the supernatant. Diameters of vesicles were measured by the dynamic light scattering analysis. The data are shown as the mean ± standard deviation from three replicates. **, $P < 0.005$ compared to WT.

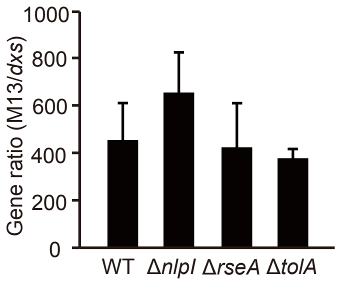

Supplementary Figure 2. Relative plasmid copy number in *E. coli* BW25113 and mutants. The ratio of pUC19 to chromosomal DNA was evaluated by real-time PCR using primers detecting M13 region and *dxs* gene, respectively. Gene content of M13/*dxs* were calculated in WT, Δ*nlpI*, Δ*rseA*, and Δ*tolA*, harboring pUC19. The data are shown as the mean ± standard deviation from three replicates.

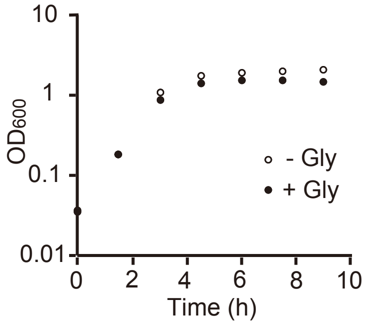

Supplementary Figure 3. Growth curves of *E. coli* with and without glycine. *E. coli* BW25113 was grown in LB medium in the absence (white) and presence (black) of 1% glycine at 200 rpm at 37°C.

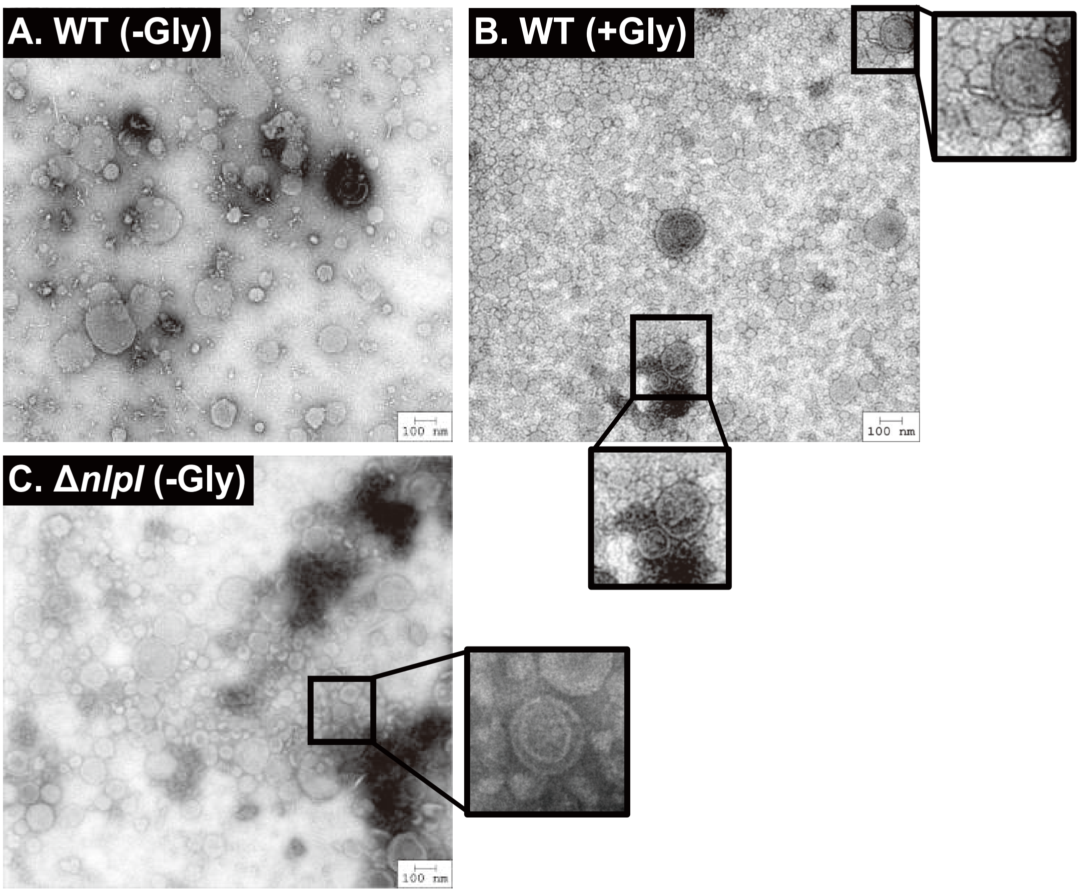

Supplementary Figure 4. Transmission electron microscopy observation of *E. coli* vesicles. *E. coli* BW25113 WT and Δ*nlpI* harboring pUC19 were grown in LB containing ampicillin with or without 1% glycine and vesicles extracted from the supernatant were observed after negative staining.

References

Baba, T., Ara, T., Hasegawa, M., Takai, Y., Okumura, Y., Baba, M., Datsenko, K.A., Tomita, M., Wanner, B.L., and Mori, H. (2006). Construction of *Escherichia coli* K-12 in-frame, single-gene knockout mutants: the Keio collection. *Mol. Syst. Biol.* 2**,** 2006.0008. doi: 10.1038/msb4100050

Datsenko, K.A., and Wanner, B.L. (2000). One-step inactivation of chromosomal genes in *Escherichia coli* K-12 using PCR products. *Proc. Natl. Acad. Sci. USA* 97**,** 6640-6645. doi: 10.1073/pnas.120163297

Ge, C., Cui, Y.-N., Jing, P.-Y., and Hong, X.-Y. (2014). An alternative suite of universal primers for genotyping in multiplex PCR. *PLoS One* 9**,** e92826. doi: 10.1371/journal.pone.0092826

Lee, C., Kim, J., Shin, S.G., and Hwang, S. (2006). Absolute and relative QPCR quantification of plasmid copy number in *Escherichia coli*. *J. Biotechnol.* 123**,** 273-280. doi: 10.1016/j.jbiotec.2005.11.014
